# Validation of a high-fidelity training model for fetoscopic spina bifida surgery

**DOI:** 10.1038/s41598-021-85607-6

**Published:** 2021-03-17

**Authors:** Luc Joyeux, Allan Javaux, Mary P. Eastwood, Felix R. De Bie, Gert Van den Bergh, Rebecca S. Degliuomini, Simen Vergote, Talita Micheletti, Geertje Callewaert, Sebastien Ourselin, Paolo De Coppi, Frank Van Calenbergh, Emmanuel Vander Poorten, Jan Deprest

**Affiliations:** 1 MyFetUZ Fetal Research Center, Department of Development and Regeneration, Cluster Woman and Child, Biomedical Sciences, Catholic University KU Leuven, Leuven, Belgium; 2 Center for Surgical Technologies, Faculty of Medicine, KU Leuven, Leuven, Belgium; 3 Department of Obstetrics and Gynecology, Division Woman and Child, Fetal Medicine Unit, University Hospitals Leuven, Leuven, Belgium; 4 Specialist Neonatal and Paediatric Surgery Unit, Great Ormond Street Hospital, University College London Hospitals, NHS Trust, London, UK; 5 Department of Mechanical Engineering, KU Leuven, Leuven, Belgium; 6 Department of Pediatric Surgery, Belfast, Northern Ireland UK; 7 Center for Fetal Diagnosis and Treatment, & the Perelman School of Medicine, the Children’s Hospital of Philadelphia, University of Pennsylvania, Philadelphia, PA USA; 8 BCNatal | Fetal Medicine Research Center, Hospital Clínic and Hospital Sant Joan de Déu, University of Barcelona, Barcelona, Spain; 9 School of Biomedical Engineering and Imaging Sciences, King’s College University, London, UK; 10 Department of Neurosurgery, University Hospitals Leuven, Leuven, Belgium; 11 Institute of Women’s Health, University College London Hospitals, London, UK

**Keywords:** Experimental models of disease, Paediatric research, Preclinical research, Translational research, Spinal cord diseases

## Abstract

Open fetal surgery for spina bifida (SB) is safe and effective yet invasive. The growing interest in fetoscopic SB repair (fSB-repair) prompts the need for appropriate training. We aimed to develop and validate a high-fidelity training model for fSB-repair. fSB-repair was simulated in the abdominal cavity and on the stomach of adult rabbits. Laparoscopic fetal surgeons served either as novices (n = 2) or experts (n = 3) based on their experience. Technical performance was evaluated using competency Cumulative Sum (CUSUM) analysis and the group splitting method. Main outcome measure for CUSUM competency was a composite binary outcome for surgical success, i.e. watertight repair, operation time ≤ 180 min and Objective-Structured-Assessment-of-Technical-Skills (OSATS) score ≥ 18/25. Construct validity was first confirmed since competency levels of novices and experts during their six first cases using both methods were significantly different. Criterion validity was also established as 33 consecutive procedures were needed for novices to reach competency using learning curve CUSUM, which is a number comparable to that of clinical fSB-repair. Finally, we surveyed expert fetal surgeons worldwide to assess face and content validity. Respondents (26/49; 53%) confirmed it with ≥ 71% of scores for overall realism ≥ 4/7 and usefulness ≥ 3/5. We propose to use our high-fidelity model to determine and shorten the learning curve of laparoscopic fetal surgeons and retain operative skills.

## Introduction

Open fetal repair for spina bifida aperta (SBA) effectively reduces postnatal morbidity^1,2^. Fetoscopic SBA repair (fSBA-repair) may minimize maternal risks and preterm delivery with similar neonatal neuroprotective effects^3–6^. However, this approach is challenging with a long learning curve (LC) of at least 56 cases^7^. A valid training model would reduce the LC and avoid or limit training on clinical subjects^8–11^. Such simulator would also accelerate the safe and ethically acceptable transition from the open approach to fetoscopy or its implementation in a center without experience based on the available case volumes. It should be paired with off- and on-site clinical training and guidance from established fetal centers^10^. Subsequently, it would enable skill retention and dissemination of the procedure^12^.

The IDEAL recommendations for surgical innovation state that preclinical studies, including simulators and valid animal models, are essential prior to first-in-human trials^12,13^. Simulators may be low-fidelity (e.g. computer simulators and inanimate bench-top trainers) or high-fidelity (e.g. in vivo animal models and human cadavers) training models. For fSBA-repair, two low-fidelity inanimate models have been proposed yet without validation^14,15^. However, they may be useful in initial training and help reducing the numbers of animals needed in further training. Two in vivo SBA models have previously been proposed for feasibility studies, i.e. fetal rhesus monkey and fetal lamb. While only the latter was used for fetoscopic surgery^16^, neither were validated^17^ or specifically designed for training. Among *smaller* animal models, mice and rats cannot be used for surgical training purposes, due to their size.

High-fidelity rather than low-fidelity surgical models enhance training realism and thus minimize potential harms from the LC in animals as well as in humans following translation to clinical practice^12,13^. Such a model for fSBA-repair requires (1) complex surgical steps like port insertion, dissection and suturing (2) simulated in a realistic environment, i.e. with proper depth perception, live motion and pulsatile blood flow in arteries and veins (3) using a living and breathing animal. Moreover, the use of large animal models should be restricted in accordance with NCR3-guidelines^18,19^. Therefore we report on the development and validation of a high-fidelity training model for fSBA-repair in a living and breathing rabbit and its use to determine the number of cases needed for a laparoscopic fetal surgeon to achieve competency.

## Methods

### Ethical statement

This experiment was approved by the Animal Ethics Committee of the Group Biomedical Sciences of the KU Leuven (P093-2016). It followed the NC3Rs and the ARRIVE guidelines for animal research^18,19^.

### Study design

The validation of this animal model followed the consensus guidelines for validation of surgical simulators^20,21^. It was assessed in two phases: construct-criterion and face-content validity^22^. For the former, the study was designed to train three laparoscopic fetal surgeons from a single fetal center, i.e. surgeons experienced in open fetal SBA repair as well as multi-port laparoscopy, yet who had never performed fSBA-repair (LJ, PDC, JD).

We categorized our surgeons into novices and experts in our model for simulated fSBA-repair based on surgical experience. Since we aimed to validate a training model and not the port-access approach, we hypothesized that one of them being a single-port and multiple-port laparoscopic neonatal surgeon was an expert (PDC). The multi-port approach is being clinical used for fetoscopic SBA repair^5,23^. By contrast, the single-port approach currently applied in fetoscopy for twin-to-twin transfusion syndrome (TTTS) or congenital diaphragmatic hernia is the ultimate minimally-invasive technique to achieve^24,25^. The other surgeons (LJ, JD) having overall less experience in multiple-port laparoscopy and no experience in single-port laparoscopy were considered as novices. They were thus trained in the model until competency in single-port fSBA-repair was reached (Supplementary Methods 1 and 2.1, Supplementary Fig. S1, Video 1). When these two surgeons had completed that training, they were then referred to as experts. In the end, these three experts performed multi-port fSBA-repair to confirm their competency (Supplementary Methods 2.2, Supplementary Fig. 1, Video 1). Overall novices and experts performed the same procedure consisting of 10 surgical steps yet used a different port-access approach (single- or multi-port).

### Description of the model

This live model was developed to mimic the operative steps and conditions present for a clinical multilayered fSBA-repair.

#### Clinical procedure to mimic

**Figure 1:**
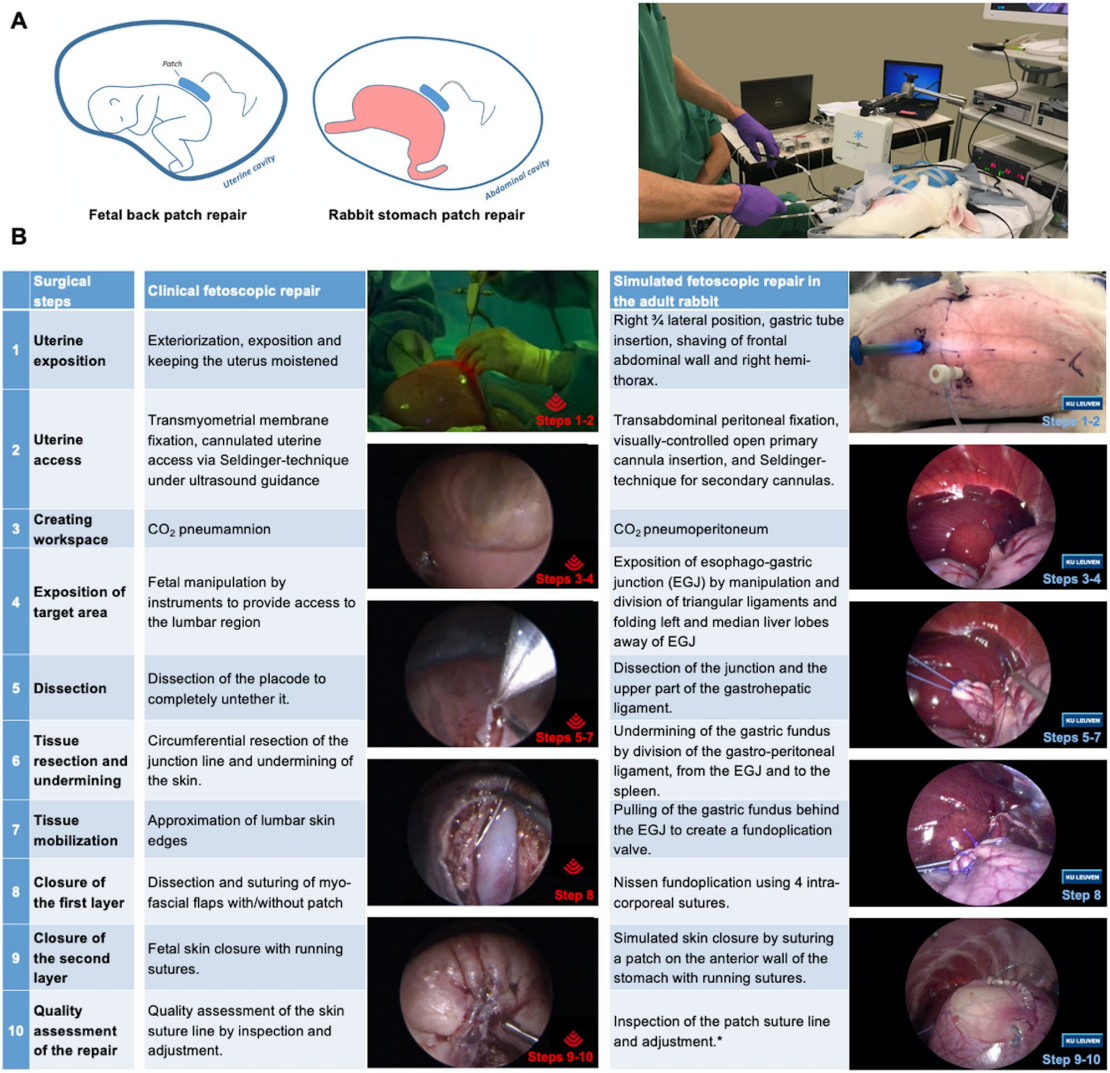
Comparison of clinical and simulated fetoscopic spina bifida repair in our rabbit training model. (**A**) Schematic drawing and external view of our model setup showing similarities in working space and presentation of the surgical target. (**B**) Comparison of the 10 essential steps performed during a clinical (left column) and simulated (right column) fetoscopic repair. Reproduced with permission of and copyright by the Texas Children’s Hospital, Houston, TX, USA and UZ Leuven. Artist drawing by Allan Javaux; copyright by UZ Leuven, Leuven, Belgium.

The gestational age at fSBA-repair in humans typically is around 24 weeks of gestation^1,5,26^. At that time the fetal weight is 662 ± 77 g^27^ and the abdominal circumference 187 ± 10 mm^27^. The region of interest is lumbar in 95% of cases^1^. The current literature on open and fSBA-repair describes several steps in the procedure, which we summarized into 10 consecutive steps to be simulated (Fig. 1)^1,5,26,28,29^.

#### Animal model

Rabbits have previously been used for training in pediatric^30,31^ and fetoscopic^32^ surgery. We used New-Zealand male adult rabbits (weight, 3-4 kg). They were given water and food at libitum under the standard light–dark cycle until the procedure. They were put under general anesthesia without endotracheal intubation (Supplementary Methods 3). In rabbits, the adequately insufflated abdominal cavity mimics the working space or amniotic cavity, which approximately measures 15 × 10x5cm with a pneumoperitoneum of around 3L with CO_2_ at 5 mmHg (Fig. 1A)^32^. Monogastric herbivores, rabbits have a large single-chamber stomach with a circumference of 165 ± 13 mm mimicking the abdominal circumference of a 22–24 weeks human fetus (Fig. 1A)^33^. Overall 10 clinical steps for fSBA-repair are recapitulated by a laparoscopic gastric Nissen fundoplication^34^ and the suturing of a patch to the gastric wall (Fig. 1B, Video 1). These procedures require the ability to gently manipulate fragile tissue, perform extensive dissection, hemostasis and suture (Supplementary Methods 3).

### Technical performance

Clinical outcomes were total operation time (steps 1 to 9)^8,9^, fetal repair time (steps 4 to 9), CO_2_ insufflation volume, Objective Structured Assessment of Technical Skill (OSATS) score and watertightness of the patch repair (step 10, Video 1). The latter was tested post-mortem by fluorescein injection under the patch after completion of the repair (Supplementary Methods 4, Video 1). Operative performance and difficulty were assessed applying an adapted OSATS rating scale on videos of the procedures (Supplementary Methods 4)^35,36^.

We also used a composite binary outcome for surgical success based on clinically relevant outcomes to measure the LC and competency level of fSBA-repair^7^. Similar to clinical fetal SBA surgery^7^, a successful surgical repair of simulated SBA was defined by a watertight repair, an operation time ≤ 180 min in accordance with the FDA Drug Safety Communication about potential risks of general anesthesia in pregnant women^37^ and an OSATS score ≥ 18/25 (> 70%)^35,36^.

### Validation study

#### Construct validity

To assess construct validity and therefore discriminate performance levels of our simulator, we determined and compared competency level of novices and experts during their first six cases applying two methods. First, the Competency Cumulative Sum (C-CUSUM) test^38^ used the aforementioned binary outcome for surgical success and was set with a control limit of h_C_ = 3 (Supplementary Methods 5.1). Subsequently, we applied the group-splitting method^9^ by comparing performance using the five aforementioned clinical outcomes.

#### Criterion validity

Criterion validity compares performance of our innovative model to the ground truth which is the clinical procedure in our case since, to the best of our knowledge, no animal training model has been validated yet^7^. Herein we compared the learning curve (LC) of novices in our model to the LC of novices performing percutaneous fSBA-repair^7^. It was determined by the predictive validity method^39^ that uses the Learning Curve CUSUM (LC-CUSUM) test with similar parameters as the C-CUSUM yet with a control limit set at h_LC_ = 0.85 (Supplementary Methods 5.2)^7^.

#### Face and content validity

To assess realism (face validity) and usefulness (content validity) of our model, an anonymous online survey (Supplementary Online Survey SurveyMonkey #6VNJMS9) was designed and sent to fetal surgeons (n = 49) worldwide who are currently involved in clinical fetal surgery programs for SBA using an open fetal and/or fetoscopic approach (Supplementary Methods 5.3, Supplementary Table S1)^7^. 22% (11/49) of the surgeons were performing fSBA-repair in their fetal center at that time. All were invited to try our model out in our research center and under our supervision to get a realistic experience. For obvious geographical reasons, some experts only answered our anonymous online questionnaire (non-users) while others also tried it (users). According to current practice, all survey responses from expert users or non-users were included in the analysis^40^. We also performed a subanalysis of data from users currently performing fetoscopic repair in humans.

Expert fetal surgeons were categorized according to their specialty (obstetricians and gynecologists, pediatric neurosurgeons and pediatric surgeons) and demographic data were captured by seven questions. We additionally asked six questions on face validity and five on content validity, using a 7-point and 5-point Likert scale respectively. We set validity thresholds at 4/7 (undecided) and 3/5 (neutral) for each scale (Supplementary Methods 5.3)^39^.

### Statistical analysis

We used GraphPad Prism version 7.00 for MacOs X (GraphPad, La Jolla, CA, USA) to analyze the data. Binomial and categorical variables were expressed as percentages with their frequency distribution. Continuous variables were tested for normal distribution using the D'Agostino-Pearson (omnibus K2) or Shapiro–Wilk normality tests. Continuous variables normally distributed were presented as mean and standard deviation (SD) and the others were expressed as median and range or interquartile range (IQR).

For face and content validity, categorical and continuous variables based on the response by the three clinical subspecialties involved in the survey were compared with one-way analysis of variance (ANOVA) or Kruskal–Wallis test as appropriate. For construct validity, the Fisher exact test was used to compare binomial variables. Continuous variables were compared with unpaired two-tailed t test or Mann Whitney test as appropriate. A p value < 0.05 was considered significant. For the construct and criterion validity, we performed C- and LC-CUSUM analysis using an algorithm that we developed in MATLAB software (Mathworks, Natick, MA, USA) based on and verified with the model of Biau et al.^7^.

## Results

### Surgical procedures

The data below were collected from 52 completed single-port simulated fSBA-repairs by two novices (n = 34 and n = 18), and from 18 multi-port simulated fSBA-repairs by three experts (n = 6, n = 6 and n = 6).

### Construct validity

**Figure 2:**
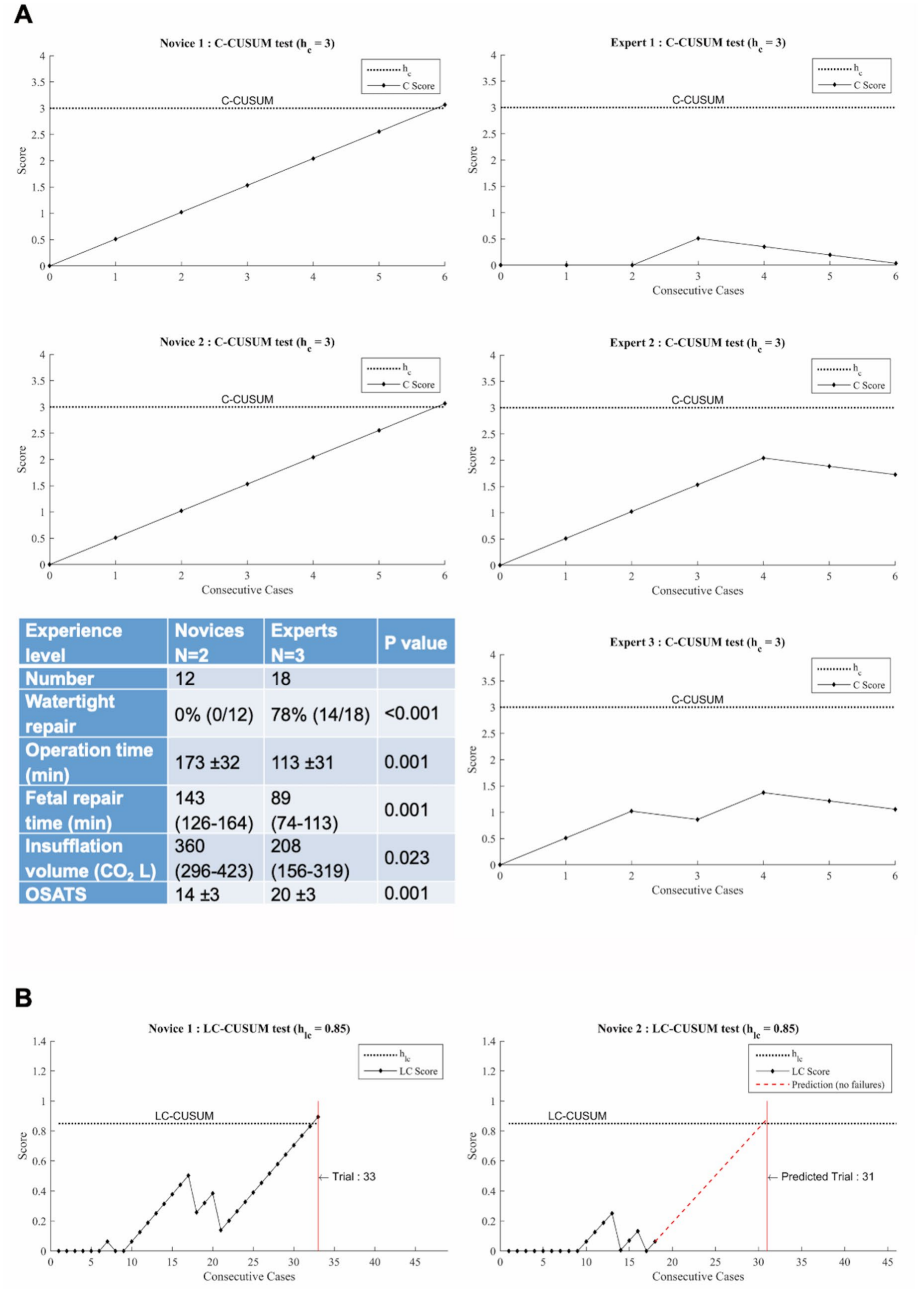
Construct and criterion validity of our training model. (**A**) Construct validity: competency assessment of novices and experts using competency cumulative sum (C-CUSUM) test (graphs), and comparison of clinical outcomes of the first six cases of novices and experts using the group splitting method (table). (**B**) Criterion validity: learning curve assessments of novices and experts using learning curve CUSUM (LC-CUSUM) test (graph).

Figure 2A displays the evolution of the C-CUSUM score of each novice and expert during the first six cases. As C-CUSUM scores of the three experts remained below the competency control limit h_C_ = 3, they were considered competent. In contrast, scores of both novices reached a higher score of 3.07 after the 6th case and were therefore not competent (graphs of Fig. 2A). The two groups were also significantly different for all surgical outcomes measured when applying the group-splitting method (table of Fig. 2A).

### Criterion validity

LC-CUSUM analysis of all the cases performed demonstrated that novice 1 reached competency when his score was 0.89, exceeding the control limit of h_LC_ = 0.85, after 33 out of 34 cases (Fig. 2B, left graph). To reduce the number of animals used, novice 2 performed only 18 procedures and did not reach that threshold. When considering the best-case scenario, it was predicted that this novice reached competency at 31 cases (Fig. 2B, right graph). These numbers involving laparoscopic fetal surgeons are lower than what has been reported as the minimum number required for competency in clinical fSBA-repair performed by non-laparoscopic fetal surgeons (n ≥ 56)^7^.

### Demographics of survey respondents

The response rate from the fetal surgeons for SBA to the online survey was 53% (26/49). 38% (10/26) of the respondents tried the model out to get a realistic experience and 27% (7/26) were performing fSBA-repair in their fetal center (Supplementary Table S2). The demographics of the different subspecialists involved were comparable, except that pediatric neurosurgeons had less experience in laparoscopic surgery (Table 1). None of the respondents was aware of computer simulators or high-fidelity models for fSBA-repair.

**Table 1:** Demographics of the three subspecialties involved in fetal surgery.

Specialty	Obstetrician and gynecologist	Pediatric neurosurgeon	Pediatric surgeon	p-value
Number	11	6	9	
**Number of years of experience as a**				
Specialist	16 ± 13	22 ± 9	17 ± 8	0.566
Laparoscopic surgeon	7 (10)	0 (1)	13 (10)	**0.003**
**Handedness**				
Right-Handed	11	5	7	
Left-Handed	0	1	2	
**Number of fetal SB open repairs in humans**				
As first surgeon	1–30 (0;1–30)	31–60 (1–30;91–120)	0 (0;31–60)	0.230
As second surgeon	1–30 (0;31–60)	1–30 (0;1–30)	1–30 (1–30;61–90)	0.363
**Number of fetal SB fetoscopic repairs in humans**				
As first surgeon	0 (0;0)	0 (0;1–30)	0 (0;1–30)	0.977
As second surgeon	0 (0;0)	0 (0;0)	0 (0;1–30)	0.779
**Number of training sessions on an open fetal surgery simulator**				
Virtual reality	0 ± 0	0 ± 0	0 ± 0	NA
Box trainer	0 (0;1–30)	0 (0;0)	0 (0;0)	0.108
Animal model	0 (0;1–30)	0 (0;0)	0 (0;31–60)	0.643
Human cadaver	0 (0;0)	0 (0;0)	0 (0;0)	0.722
**Number of training sessions on a fetoscopic surgery simulator**				
Virtual reality	0 ± 0	0 ± 0	0 ± 0	NA
Box trainer	0 (0;1–30)	1–30 (0;1–30)	0 (0;1–30)	0.449
Animal model	0 (0;1–30)	0 (0;1–30)	0 (0;1–30)	0.648
Human cadaver	0 ± 0	0 ± 0	0 ± 0	NA

Number of surgeries per specialty are displayed in median and range based on 5 block of 30 cases as per the online survey (0, 1–30, 31–60, 61–90, 91–120, ≥ 121). For example 31–60 (1–30;91–120) signifies that a mean of 1–30 cases was performed and the number of cases varied between 1–30 and 91–120.

### Face validity

The three subspecialties of respondents considered our live model realistic since all survey questions reached scores ≥ 4/7 in ≥ 60% of cases and recommended it for its realism (≥ 84% of scores ≥ 4/7; Table 2). Good to exceptional scores (63 to 100% of scores ≥ 4/7) were reached for questions that required a living and breathing animal. These encompassed the surgical target, surgical steps such as dissection, resection and suturing, and depth perception (Table 2). Despite some discrepancies, there were no significant differences in ratings between the subspecialists. In addition, the seven fetoscopic experts who used our model confirmed its realism as 95% of survey questions scored ≥ 4/7. They also recommended it for its realism (100% of scores ≥ 4/7; Supplementary Table S3).

**Table 2:** Face and content validity of the model.

Subspecialty	Obstetrician and gynecologist	Pediatric neurosurgeon	Pediatric surgeon	P value
Number	11	6	9	

Face validity	Percentages of scores ≥ 4/7 on the Likert scale			
**Overall realism**				
Recommendation for realism	88%	94%	84%	0.874
**Surgical scene**				
Animal positioning	80%	83%	75%	0.916
Position of the video monitor	100%	100%	100%	0.216
Position of 1st surgeon	100%	100%	88%	0.557
Position of 2nd surgeon	100%	100%	75%	0.933
**Surgical cavity**				
Humidified environment (Fluid-gas interface)	90%	100%	88%	0.804
Workspace	90%	100%	88%	0.034*
Vision	100%	100%	88%	0.419
**Surgical target**				
Mimic of the fetal lumbar region	70%	84%	73%	0.961
**Instrumentation set**				
Endoscope	90%	100%	100%	0.440
Grasping forceps	90%	100%	100%	0.253
Scissors	100%	100%	100%	0.648
Coagulating and dissecting hook	100%	100%	100%	0.646
Dissector	100%	100%	100%	0.349
Needle holders	100%	100%	100%	0.167
**Surgical steps**				
Exposition	80%	83%	75%	0.750
Port insertion	60%	83%	88%	0.201
Insufflation	100%	100%	100%	0.753
Fetal positioning	70%	83%	63%	0.834
Dissection	70%	83%	63%	0.964
Resection	70%	83%	63%	0.889
Mobilization	80%	100%	63%	0.984
Patch	90%	83%	63%	0.521
Skin	90%	83%	75%	0.159
Quality assessment	100%	83%	88%	0.507
**Depth perception**				
Mimic of clinical conditions (live motions)	80%	100%	100%	0.110
**Content validity**	**Percentages of scores ≥ 3/5 on the Likert scale**			
**Overall usefulness**				
Recommendation for training	80%	83%	71%	0.332
**Overall difficulty**				
As difficult as in humans	80%	100%	71%	0.815
Similar stress	50%	50%	86%	0.104
**Instrument handling**				
Improvement of instrument handling skills	100%	83%	100%	0.690
**Suturing**				
Improvement of suturing skills	100%	100%	100%	0.832
**Self-confidence**				
Overall confidence	100%	100%	100%	0.180
**Surgical Tasks**				
Exposition	80%	83%	71%	0.501
Port insertion	90%	83%	71%	0.568
Insufflation	100%	83%	100%	0.718
Fetal positioning	60%	83%	71%	0.712
Dissection	80%	83%	71%	0.595
Resection	80%	83%	71%	0.642
Mobilization	80%	83%	86%	0.829
Patch	90%	83%	71%	0.939
Skin	90%	83%	100%	0.385
Quality assessment	100%	83%	100%	0.406

There were no significant differences between each of the 3 specialties of fetal surgeons.

### Content validity

There were no significant differences in ratings between the subspecialists. Respondents considered the model useful and would recommend it for training (≥ 71% of scores ≥ 3/5) and improving complex fetoscopic skills, such as instrument handling and suturing (≥ 83% and 100% of score ≥ 3/5 respectively; Table 2). Average (≥ 50%) scores varied among the three subspecialties and were obtained when we asked whether the model exposes to stress similar to that in clinical conditions and is useful to train for fetal positioning (≥ 50% and ≥ 60% of scores ≥ 3/5 respectfully). In contrast high scores (≥ 83% of scores ≥ 3/5) concerned the usefulness of the model for instrument handling, suturing, self-confidence, insufflation, tissue mobilization, skin closure and quality assessment of the patch repair. Finally, the seven fetoscopic experts who used our model confirmed its usefulness as 98% of survey questions scored ≥ 3/5. They also recommended it for its training (100% of scores ≥ 3/5; Supplementary Table S3).

## Discussion

### Main findings

We developed and validated a high-fidelity training model for fetoscopic SBA repair in live rabbits. We first demonstrated that competency of laparoscopic fetal surgeons was reached at 33 consecutives cases. That number is lower than what has been reported for non-laparoscopic fetal surgeons performing clinical multi-port fSBA-repair. Surveyed fetal experts also proved face and content validity.

### Clinical interpretation

A recent systematic review with available individual patient data on multi-port fSBA-repair demonstrated that the LC to reach competency was at least 56 cases^7^. This is of the same order of magnitude of other complex multi-port laparoscopic surgeries such as colectomy^41,42^ or sacrocolpopexy^43^ performed by surgeons without previous experience in these techniques. In our high-fidelity model the LC was 33 cases for novices, more precisely surgeons experienced in multi-port laparoscopic surgery but non-experienced with single-port surgery. That number is in keeping with other complex single-port procedures such as colectomy performed by surgeons experienced in multi-port laparoscopic surgery^44^. It is also similar to other advanced, yet less complex multi-port endoscopic procedures than those above, such as cholecystectomy^45^, pyloromyotomy^46^ or the most common fetoscopic operation (laser coagulation for TTTS)^24^. They are described as less complex as they do not require suturing and extensive dissection (or in case of TTTS none at all) skills. We therefore surmise that the number of 33 reached in our simulator is the LC of surgeons experienced with complex multi-port laparoscopy before translation to clinical practice. It may represent an underestimation for surgeons non-experienced with complex multi-port laparoscopy. Clinically, the challenges of fetal surgery are greater than what can be simulated, such as the complex pathologic anatomy of the lesion, the frailty of human fetal tissue, the interference of fetal monitoring, the presence and vicinity of the placenta, the large number of people and specialties around the operation table, or simply the stress of operating on two patients. These points were suggested by 6/26 (23%) of the survey participants (3/6 pediatric neurosurgeons, 2/9 pediatric surgeons and 1/11 obstetrician and gynecologist).

### Strength and limitations

We acknowledge a number of limitations to our study. First, some surveyed fetal surgeons raised concerns regarding the realism of specific aspects of our model. Indeed, the simulation procedure does not mimic the precise dissection and gentle manipulation of the dura, musculo-fascial flaps and fetal skin. However, manipulation, dissection and suturing of the stomach are quite comparable to these clinical steps, as the rabbit stomach can be easily damaged and perforated. This way, those steps unmask potential clinically relevant complications. Secondly, three laparoscopic fetal surgeons were involved in our fetal surgery training to reduce the number of animal required hence following the ethical standards of the NC3Rs-guidelines^18,19^. Our competency analysis allowed us to confirm our hypothesis about the competency level of novices and experts. Since experts 1 and 2 were previously novices 1 and 2, these surgeons became experts in three-port surgery in our rabbit training model—yet not for clinical fSBA-repair—following their training as novices in single-port surgery. Finally, we only tested our model for either a single- and three-port^23^. A two-port approach^26^ currently practiced by some centers can easily be adapted.

Our study also has considerable strengths. Firstly, in the development of a simulator, we followed the consensus guidelines for animal research and validation of animal models^18,39^ and surgical simulators^13,14,22^. Secondly, we applied robust methods for assessing subjective and objective validity. Thirdly, we measured the LC and competency level of both experts and novices with standardized methodology^38^. Finally, our observations seem clinically relevant as we come to numbers that are comparable to what has been demonstrated for complex clinical laparoscopic procedures performed by trained laparoscopic surgeons.

## Conclusion

We developed and validated a high-fidelity model for fetoscopic layered SBA repair. It was used to determine the learning curve of laparoscopic fetal surgeons, which was in the range of other complex endoscopic procedures. We propose the use of this model to determine and shorten the learning curve of laparoscopic fetal surgeons, and aid retention of operative skills.

## Supplementary Information

Supplementary Video 1.

Supplementary Information 1.

Supplementary Information 2.
