# Supplementary material for: Validation of a high-fidelity training model for fetoscopic spina bifida surgery
Source: Sci Rep. 2021 Mar 17;11:6109. doi: 10.1038/s41598-021-85607-6 (PMC7969952; doi:10.1038/s41598-021-85607-6)
Supplement: Supplementary file 2 — Supplementary Information 1. [file 41598_2021_85607_MOESM2_ESM.docx]

**Supplemental digital content**

**Title**

**Validation of a high-fidelity training model for fetoscopic spina bifida surgery**

**Authors**

Luc Joyeux^1-4^*, Allan Javaux^5^*, Mary P. Eastwood^1,2,6^, Felix De Bie^1,2^, Gert Van den Bergh^2^, Rebecca S. Degliuomini^1^, Simen Vergote^1,2^, Talita Micheletti^1,8^, Geertje Callewaert^1-3^, Sebastien Ourselin^9^, Paolo De Coppi^1,4^, Frank Van Calenbergh^10^, Emmanuel Vander Poorten^5^, Jan Deprest^1-3,11^

**Affiliations**

1. MyFetUZ Fetal Research Center, Department of Development and Regeneration, Cluster Woman and Child, Biomedical Sciences, Catholic University KU Leuven, Leuven, Belgium
2. Center for Surgical Technologies, Faculty of Medicine, KU Leuven, Leuven, Belgium
3. Department of Obstetrics and Gynecology, Division Woman and Child, Fetal Medicine Unit, University Hospitals Leuven, Leuven, Belgium
4. Specialist Neonatal and Paediatric Surgery Unit, Great Ormond Street Hospital, University College London Hospitals, NHS trust, London, United Kingdom
5. Department of Mechanical Engineering, KU Leuven, Leuven, Belgium
6. Department of Pediatric Surgery, Belfast, Northern Ireland, United Kingdom
7. Center for Fetal Diagnosis and Treatment, the Children’s Hospital of Philadelphia, & the Perelman School of Medicine, University of Pennsylvania, Philadelphia, PA, USA
8. BCNatal | Fetal Medicine Research Center, Hospital Clínic and Hospital Sant Joan de Déu, University of Barcelona, Barcelona, Spain
9. School of Biomedical Engineering and Imaging Sciences, King’s College University, London, United Kingdom
10. Department of Neurosurgery, University Hospitals Leuven, Leuven, Belgium
11. Institute of Women’s Health, University College London Hospitals, London, United Kingdom

* These authors contributed equally to this work." Both authors contributed equally to this work.

**Corresponding authors**

Luc Joyeux, MD MSc and Jan Deprest, MD PhD

MyFetUZ Fetal Research Center, Department Development and Regeneration, Cluster Woman and Child, Biomedical Sciences, KU Leuven, Leuven, Belgium.

UZ Herestraat 49, box 1034, 3000 Leuven, Belgium

E-Mail: [luc.joyeux@kuleuven.be](mailto:luc.joyeux@kuleuven.be) and [jan.deprest@uzleuven.be](mailto:jan.deprest@uzleuven.be)

**Keywords:** Spina bifida, myelomeningocele, fetal surgery, fetoscopy, training model, high-fidelity, rabbit, validation, learning curve.**Supplementary methods**

1. **Study design**

Both laparoscopic fetal surgeons unexperienced with single-port fetoscopy first completed an intensive purposely designed single-port training program (≥10 hours) on a bench-top SILS simulator and up to three rabbit cadavers.[^1^](#_ENREF_1) This training program consisted of LASTT (Laparoscopic Skills Training and Testing method)[^2^](#_ENREF_2) and E-BLUS (European Basic Laparoscopic Urological Skills examination)[^3^](#_ENREF_3) exercises on a pelvic endotrainer (Ethicon) adapted to use with a single-port system (GelPOINT Mini Advanced Access Platform, Applied Medical, Amersfoort, the Netherlands). Basic exercises had to be done three consecutive times: LASTT exercise 1 for camera handling in <120s, exercise 2 for hand-eye coordination (single-handed placing of rings on pins, both with non-dominant and dominant hand) in <180s and exercise 3 for bi-manual coordination (handling of pins) in <180s; E-BLUS exercise 1 for bi-manual peg transfers in <126s), exercise 2 for pattern cutting of a circle from a gauze in <151s, exercise 3 for intracorporeal single knot tying with non-dominant and dominant hand on a Penrose drain in <360s and exercise 5 for needle guidance through 10 metal rings following a set route in <268s.

Then the three surgeons were allowed up to six training sessions of around three hours on either a rabbit cadaver (n=3) or in vivo (n=3) while standardizing their single-port simulated procedure. They additionally had three in vivo rabbit trainings to standardize the three-port procedure (Supplementary Figure 1B).

1. **Instrumentation**
   1. Equipment for single-port endoscopy

A purposely-made 5mm single-port instrumentation set was kindly provided by Karl Storz (Tuttlingen, Germany). It had the complete single-port LEROY S-PORT system including a port ring (23030P) with four 5mm seals (23030SA), an attachment tool (23030X), an insertion aid tool (23030D) and a removal tool (23030T). Further it contained the 5mm straight rod lens endoscope (45° angle of view, 60cm length, 26048 FSA) and seven curved instruments (Supplementary Figure 1A).

- 1. Equipment for three-port endoscopy

A 3mm 3-port pediatric instrumentation set was provided by Karl Storz. It included a 5mm straight scope (TIPCAM1 S, 30° angle, 4mm diameter, 18 cm length, 7240 BA3D) inserted in a 5mm port with a balloon (5mmx100mm Kii Advanced Fixation sleeve trocar, CFR03, Applied Medical, Rancho Santa Margarita, CA, USA). Seven 3mm 20cm long laparoscopy instruments were alternatively inserted into two 4mm ports (12-Fr Check-Flo Performer, 13cm length, RCF-12.0-38-J, Cook Medical Inc., Bloomington, IN, USA) (Supplementary Figure 1B).

1. **Description of the model**

New-Zealand male rabbits were provided by the university farm and fed with water and food ad libitum under a usual light-dark cycle. They underwent a standardized general anesthesia without intubation. First, the animal was sedated with xylazine (6mg/kg IM; Xyl-M 2%, VMD, Arendonk, Belgium) and ketamine (35mg/kg IM; Nimatek 100 mg/ml, Eurovet Animal Health BV, Bladel, the Netherlands) and then weighed. Second, after insertion of a gastric tube (enteral feeding tube 50cm-8Fr-PVC, 310.08, Vygon, Ecouen, France), 1-2% isoflurane (Iso-Vet 1000mg/g, Dechra, Lille, Belgium) in 100% oxygen at 2L/min was administered for maintenance via a breathing mask. Third, they were injected with an analgesic, buprenorphine (0.03mg/kg SC; Vetergesic 0,3 mg/ml, Ecuphar, Oostkamp, Belgium).

The operation consisted in 10 steps, of which eight for the gastric fundoplication. Once exposition and dissection of the esophagogastric junction was accomplished, the surgeon dissected the posterior wall of the esophagus and the gastric fundus to create a 360° anti-reflux fundus valve and sutured the valve with 4 interrupted sutures (4-0 Prolene RB-1, BP8557, Ethicon, Somerville, NJ, USA). Thereafter two additional steps included the suturing of a prosthetic 3x3cm patch (Integra dermal regeneration template, Integra Life Sciences, Plainsboro, NJ, USA) to the anterior wall of the stomach was performed by two half circle running sutures (4-0 V-Loc 180 CV-23, VLOCL0803, Medtronic, Dublin, Ireland).

1. **Technical performance**

After completion of the procedure, the rabbits were euthanized using IV 0.5mL/kg barbiturate (Dolethal, Vetoquinol, Aartselaar, Belgium) and installed dorsal decubitus. Their abdomen was opened to expose the entire stomach and assess the watertightness suturing of the patch using diluted fluorescein (Minims Fluorescein Sodium 2% w/v, Bausch & Lomb pharma, Laval, Quebec, Canada).[^4^](#_ENREF_4) We diluted 0.1 mL fluorescein in 4 mL saline thus obtaining a dilution 20-times lower than the one usually used in neurosurgery.[^4^](#_ENREF_4) A microcatheter (BPU-T30, polyutherane tube 3Fr, Solomon Scientific, Harvard Apparatus, Les Ulis, France) was inserted under the patch through the stomach wall using a 18 Ga catheter (BD Insyte-W, 381346, Franklin Lakes, New Jersey, USA). Diluted fluorescein was injected at a standardized pressure of 30cm H_2_O similar to the highest CSF pressure measured in children.[^5^](#_ENREF_5) The mixture was then visualized with a 51-LED 395nm-ultraviolet flashlight (Oxyled, Shenzhen Thousand Shores Technology Co Ltd, Shenzhen, China). Watertightness was determined by two independent observers at the end of the surgery, as the absence of any leakage of fluorescein between the suture runs.

Operative performance and difficulty were assessed applying the OSATS rating scale on videos of the procedures (Supplementary Methods 4).[^6^](#_ENREF_6)^,^[^7^](#_ENREF_7) Scores were determined by three independent raters blinded to the surgeon. We adapted the OSATS score to a maximum score of 25 instead of 35.[^7^](#_ENREF_7) We could not include the last two out of seven items, i.e. use of assistants and knowledge of specific procedure, since each surgeon had the same assistant and learned the 10 steps before starting the training. The first five items of the scale were consequently used: respect for tissue, time and motion, instrument handling, instrument knowledge and flow of operation.

1. **Validation study**
   1. Construct validity

In surgery, construct validity refers to the degree to which the simulator can discriminate performance levels. To assess it, we determined and compared competency level of novices and experts during their first six cases applying two methods.

First, we applied the Competency Cumulative Sum (C-CUSUM) test[^8^](#_ENREF_8) which statistically concludes if a surgeon remains competent - retain his surgical skills - based on the accumulation of failures. The following parameters are required: adequate performance (i.e. expected probability of failure for a competent surgeon), inadequate performance (i.e. minimum expected probability of failure for incompetent surgeon), and the statistical limit of the test. The first two parameters where chosen by a consensus and literature review.[^7^](#_ENREF_7) The last one was determined such that the error rates of the C-CUSUM test (true negatives and false positives) for the chosen adequate and inadequate performance are optimized. This was done through simulations of 10,000 samples.[^8^](#_ENREF_8) We used the binary outcome for surgical success mentioned in the Methods-Technical performance section. Similar to clinical fetal SBA surgery, the range of an adequate, i.e. clinically acceptable, failure rate was based on the MOMS trial and thus was set at 13-30%.[^7^](#_ENREF_7)^,^[^9^](#_ENREF_9) Adequate and inadequate performance were consequently considered when the failure leakage rate was ≤18% and ≥30% respectively. We considered a 5% acceptable deviation (delta) and control limit of h_C_=3.[^7^](#_ENREF_7)^,^[^8^](#_ENREF_8) In brief, for each failure the surgeon’s score would increase of 0.511 and for each success it would drop of 0.158 (the minimum cumulative score being 0).

Subsequently, we applied the group-splitting method[^10^](#_ENREF_10) by comparing performance using the five aforementioned clinical outcomes.

- 1. Criterion validity

Criterion validity compares performance of our model to the ground truth which is the clinical procedure in our case.[^7^](#_ENREF_7) Herein we compared the learning curve (LC) of novices in our model to the LC of novices performing percutaneous fetoscopic SBA repair. To assess their LC and conclude how many cases were necessary to achieve competency, we applied the predictive validity method[^11^](#_ENREF_11) with the Learning Curve CUSUM (LC-CUSUM) test[^12^](#_ENREF_12). The same parameters as for the C-CUSUM test were used but adding one parameter, i.e. acceptable deviation from performance, which is typically half the distance between adequate and inadequate performance.[^12^](#_ENREF_12) The computation of the statistical limit was redone through simulations of another 10,000 samples and the control limit was set at h_LC_=0.85.[^7^](#_ENREF_7) Should that threshold not be reached within all experiments of one surgeon, we could say that the surgeon had not reached competency. In such case, a linear interpolation was performed using the best-case scenario[^7^](#_ENREF_7) to predict the number of cases required to reach competency and therefore reduce the number of animals used. The best-case scenario considers each new potential case as a success until the LC-CUSUM score is above h_LC_. In summary, for each success the surgeon’s score would increase of 0.063 and for each failure it would drop of 0.245, the minimum cumulative score being 0.

- 1. Face and content validity

Face validity refers to the degree of resemblance (further referred to as “realism”) between the simulated and the clinical procedure as determined by experts in the field. Content validity examines whether experts agree the live model to be appropriate and useful for teaching the procedure. Both tests should be based on “the judgements of researchers and health care professionals as well as input from service users”.[^13^](#_ENREF_13) Therefore we contacted fetal surgeons (experts) worldwide who are currently involved in clinical fetal surgery programs for SBA using an open fetal and/or fetoscopic approach. We emailed an anonymous online survey to each surgeon and expected a response following a usual 3-month period of three rounds of invitation letters. Moreover all experts were invited to try our model. For obvious geographical reasons, some experts only answered our anonymous online questionnaire (researchers) while others also tried it (service users). All survey responses from experts (users or non-users) were included in the analysis.[^13^](#_ENREF_13) We also performed a subanalysis of data from users currently performing fetoscopic repair in humans.

The datasets generated during and/or analyzed during the current study are available from the corresponding author on reasonable request.

Supplementary tables and figures

***Supplementary Table 1 -*** *List of fetal surgeons surveyed and their fetal surgery centers.*

| **Name** | **Initial** | **Center** | **Country** |
| --- | --- | --- | --- |
| Acacio | G. | Sao Paulo | Brazil |
| Adzick | S.N. | Philadelphia | USA |
| Alireza | A.S. | Houston | USA |
| Basurto | D. | Leuven | Belgium |
| Belfort | M. | Houston | USA |
| Bennett | A.K. | Nashville | USA |
| Black | L. | Nashville | USA |
| Blazquez | M. | Zurich | Switzerland |
| Bohosiewicz | J. | Bytom | Poland |
| Carreras | E. | Barcelona | Spain |
| Cavalheiro | S. | Sao Paulo | Brazil |
| Danzer | E. | Philadelphia | USA |
| De Catte | L. | Leuven | Belgium |
| De Coppi | P. | London | UK |
| Deprest | J. | Leuven | Belgium |
| Devlieger | R. | Leuven | Belgium |
| Elbabaa | S.K. | Saint Louis | USA |
| Farmer | D.L. | San Francisco | USA |
| Flake | A. | Philadelphia | USA |
| Heuer | G.G. | Philadelphia | USA |
| Hirose | S. | San Francisco | USA |
| Johnson | M.P. | Philadelphia | USA |
| Jouannic | J.M. | Paris | France |
| Joyeux | L. | Leuven | Belgium |
| Keswani | S.G. | Houston | USA |
| King | A. | Houston | USA |
| Kohl | T. | Giessen | Germany |
| Lapa | D.A. | Sao Paulo | Brazil |
| Lopez | M. | Barcelona | Spain |
| Mazzone | L. | Zurich | Switzerland |
| Meier | J. | Zurich | Switzerland |
| Meuli | M. | Zurich | Switzerland |
| Moehrlen | U. | Zurich | Switzerland |
| Moldenhauer | J. | Philadelphia | USA |
| Moron | A.F. | Sao Paulo | Brazil |
| Ochsenbein | N. | Zurich | Switzerland |
| Olejek | A. | Bytom | Poland |
| Peralta | F. | Sao Paulo | Brazil |
| Peranteau | W. | Philadelphia | USA |
| Ryan | G. | Toronto | Canada |
| Thompson | D. | London | UK |
| Valenzuela | I. | Leuven | Belgium |
| Van Calenbergh | F. | Leuven | Belgium |
| Van Mieghem | T. | Toronto | Canada |
| Vlastos | E.J. | Saint Louis | USA |
| Weiner | H.L. | Houston | USA |
| Whitehead | W.E. | Houston | USA |
| Zerah | M. | Paris | France |
| Zimmermann | R. | Zurich | Switzerland |

***Supplementary Table 2 – Demographics of the 7 fetoscopic surgeons that tried the model.*** *Number of surgeries per specialty are displayed in median and range based on 5 block of 30 cases as per the online survey (0, 1-30, 31-60, 61-90, 91-120, ≥121).*

| **Specialty** | **Obstetrician and gynecologist** | **Pediatric neurosurgeon** | **Pediatric surgeon** |
| --- | --- | --- | --- |
| **Number** | 2 | 2 | 3 |
| **Number of years of experience as a** | | | |
| **Specialist** | 7 & 38 | 23 & 16 | 17 & 18 & 10 |
| Laparoscopic surgeon | 7 & 30 | 0 & 5 | 13 & 12 & 10 |
| **Handedness** | | | |
| **Right-Handed** | 2 | 2 | 1 |
| **Left-Handed** | 0 | 0 | 2 |
| **Number of fetal SB open repairs in humans** | | | |
| As first surgeon | 1-30 & 31-60 | 0 & 31-60 | 0 & 0 & 0 |
| As second surgeon | 31-60 & 31-60 | 1-30 & 0 | 0 & 0 & 1-30 |
| **Number of fetal SB fetoscopic repairs in humans** | | | |
| As first surgeon | 1-30 & 1-30 | 0 & 31-60 | 1-30 & 1-30 & 0 |
| As second surgeon | 31-60 & 31-60 | 1-30 & 0 | 1-30 & 1-30 & 1-30 |
| **Number of training sessions on an open fetal surgery simulator** | | | |
| Virtual reality | 0 & 0 | 0 & 0 | 0 & 0 & 0 |
| Box trainer | 1-30 & 0 | 0 & 0 | 0 & 0 & 0 |
| Animal model | 1-30 & 0 | 0 & 0 | 0 & 0 & 0 |
| Human cadaver | 0 & 0 | 0 & 0 | 0 & 0 & 0 |
| **Number of training sessions on a fetoscopic surgery simulator** | | | |
| Virtual reality | 0 & 0 | 0 & 0 | 0 & 0 & 0 |
| Box trainer | 1-30 & 61-90 | 1-30 & 1-30 | 1-30 & 1-30 & 0 |
| Animal model | 1-30 & 1-30 | 1-30 & 61-90 | 1-30 & 1-30 & 1-30 |
| Human cadaver | 0 & 0 | 0 & 0 | 0 & 0 & 0 |

***Supplementary Table 3 - Face and content validity of the model.***

| **Subspecialty** | **Obstetrician and gynecologist** | **Pediatric neurosurgeon** | **Pediatric**  **surgeon** |
| --- | --- | --- | --- |
| **Number** | **2** | **2** | **3** |
| **Face validity** | **Percentages of scores ≥4/7 on the Likert scale** | | |
| **Overall realism** |  |  |  |
| Recommendation for realism | 100% | 100% | 100% |
| **Surgical scene** |  |  |  |
| Animal positioning | 100% | 100% | 67% |
| **Position of the video monitor** | 100% | 100% | 100% |
| Position of 1^st^ surgeon | 100% | 100% | 67% |
| Position of 2^nd^ surgeon | 100% | 100% | 67% |
| **Surgical cavity** |  |  |  |
| Humidified environment  (Fluid-gas interface) | 100% | 100% | 100% |
| Workspace | 100% | 100% | 100% |
| Vision | 100% | 100% | 100% |
| **Surgical target** |  |  |  |
| Mimic of the fetal lumbar region | 100% | 100% | 100% |
| **Instrumentation set** |  |  |  |
| **Endoscope** | 100% | 100% | 100% |
| **Grasping forceps** | 100% | 100% | 100% |
| **Scissors** | 100% | 100% | 100% |
| **Coagulating and dissecting hook** | 100% | 100% | 100% |
| **Dissector** | 100% | 100% | 100% |
| **Needle holders** | 100% | 100% | 100% |
| **Surgical steps** |  |  |  |
| **Exposition** | 100% | 100% | 100% |
| **Port insertion** | 100% | 100% | 100% |
| **Insufflation** | 100% | 100% | 100% |
| **Fetal positioning** | 100% | 100% | 67% |
| **Dissection** | 100% | 100% | 100% |
| **Resection** | 100% | 100% | 100% |
| **Mobilization** | 100% | 100% | 100% |
| **Patch** | 100% | 100% | 100% |
| **Skin** | 100% | 100% | 100% |
| **Quality assessment** | 100% | 100% | 100% |
| **Depth perception** |  |  |  |
| **Mimic of clinical conditions**  **(live motions)** | **100%** | **100%** | **100%** |
| **Content validity** | **Percentages of scores ≥3/5 on the Likert scale** | | |
| **Overall usefulness** |  |  |  |
| Recommendation for training | **100%** | **100%** | **100%** |
| **Overall difficulty** |  |  |  |
| **As difficult as in humans** | **100%** | **100%** | **100%** |
| Similar stress | **100%** | **100%** | **100%** |
| **Instrument handling** |  |  |  |
| Improvement of instrument handling skills | **100%** | **100%** | **100%** |
| **Suturing** |  |  |  |
| Improvement of suturing skills | **100%** | **100%** | **100%** |
| **Self-confidence** |  |  |  |
| **Overall confidence** | **100%** | **100%** | **100%** |
| **Surgical Tasks** |  |  |  |
| **Exposition** | **100%** | **100%** | **100%** |
| **Port insertion** | **100%** | **100%** | **100%** |
| **Insufflation** | **100%** | **100%** | **100%** |
| **Fetal positioning** | **50%** | **100%** | **100%** |
| **Dissection** | **100%** | **100%** | **100%** |
| **Resection** | **100%** | **100%** | **100%** |
| **Mobilization** | **100%** | **100%** | **100%** |
| **Patch** | **100%** | **100%** | **100%** |
| **Skin** | **100%** | **100%** | **100%** |
| **Quality assessment** | **100%** | **100%** | **100%** |

***Supplementary Figure 1*** *– Surgical instrumentation for simulated spina bifida aperta fetoscopic repair. Single- (A) and three-port (B) sets. Pictures by K. Storz and copyright by UZ Leuven, Belgium.*


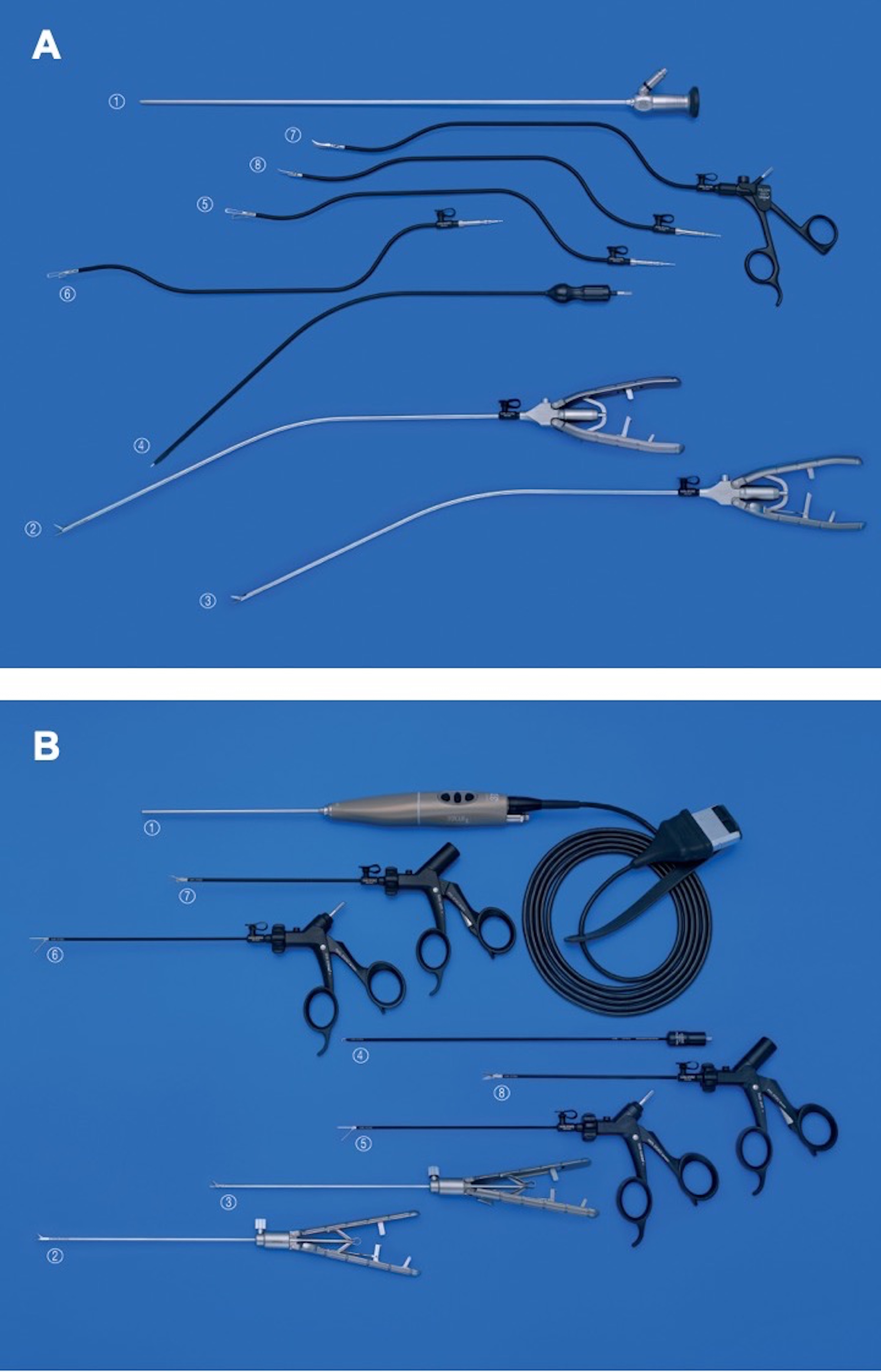


**REFERENCES**

1 Brown-Clerk, B. *et al.* Laparoendoscopic single-site (LESS) surgery versus conventional laparoscopic surgery: comparison of surgical port performance in a surgical simulator with novices. *Surg Endosc* **25**, 2210-2218, doi:10.1007/s00464-010-1524-x (2011).

2 Campo, R. *et al.* A valid model for testing and training laparoscopic psychomotor skills. *Gynecological Surgery* **7**, 133-141, doi:10.1007/s10397-009-0547-6 (2010).

3 Brinkman, W. M. *et al.* Results of the European Basic Laparoscopic Urological Skills examination. *European urology* **65**, 490-496, doi:10.1016/j.eururo.2013.10.036 (2014).

4 Prosser, J. D., Vender, J. R. & Solares, C. A. Traumatic cerebrospinal fluid leaks. *Otolaryngologic clinics of North America* **44**, 857-873, vii, doi:10.1016/j.otc.2011.06.007 (2011).

5 Avery, R. A. *et al.* Reference range for cerebrospinal fluid opening pressure in children. *N Engl J Med* **363**, 891-893, doi:10.1056/NEJMc1004957 (2010).

6 Martin, J. A. *et al.* Objective structured assessment of technical skill (OSATS) for surgical residents. *The British journal of surgery* **84**, 273-278, doi:10.1046/j.1365-2168.1997.02502.x (1997).

7 Joyeux, L. *et al.* Learning curves of open and endoscopic fetal spina bifida closure: systematic review and meta-analysis. *Ultrasound in obstetrics & gynecology : the official journal of the International Society of Ultrasound in Obstetrics and Gynecology* **55**, 730-739, doi:10.1002/uog.20389 (2020).

8 Biau, D. J. & Porcher, R. A method for monitoring a process from an out of control to an in control state: Application to the learning curve. *Stat Med* **29**, 1900-1909, doi:10.1002/sim.3947 (2010).

9 Adzick, N. S. *et al.* A randomized trial of prenatal versus postnatal repair of myelomeningocele. *N Engl J Med* **364**, 993-1004, doi:10.1056/NEJMoa1014379 (2011).

10 Khan, N., Abboudi, H., Khan, M. S., Dasgupta, P. & Ahmed, K. Measuring the surgical 'learning curve': methods, variables and competency. *Bju Int* **113**, 504-508, doi:10.1111/bju.12197 (2014).

11 Boateng, G. O., Neilands, T. B., Frongillo, E. A., Melgar-Quinonez, H. R. & Young, S. L. Best Practices for Developing and Validating Scales for Health, Social, and Behavioral Research: A Primer. *Front Public Health* **6**, 149, doi:10.3389/fpubh.2018.00149 (2018).

12 Biau, D. J., Williams, S. M., Schlup, M. M., Nizard, R. S. & Porcher, R. Quantitative and individualized assessment of the learning curve using LC-CUSUM. *The British journal of surgery* **95**, 925-929, doi:10.1002/bjs.6056 (2008).

13 Connell, J. *et al.* The importance of content and face validity in instrument development: lessons learnt from service users when developing the Recovering Quality of Life measure (ReQoL). *Quality of life research : an international journal of quality of life aspects of treatment, care and rehabilitation* **27**, 1893-1902, doi:10.1007/s11136-018-1847-y (2018).
