# Supplementary material for: Validation of a high-fidelity training model for fetoscopic spina bifida surgery
Source: Sci Rep. 2021 Mar 17;11:6109. doi: 10.1038/s41598-021-85607-6 (PMC7969952; doi:10.1038/s41598-021-85607-6)
Supplement: Supplementary file 3 — Supplementary Information 2. [file 41598_2021_85607_MOESM3_ESM.docx]

**Evaluation of a high-fidelity simulation model**

**for fetoscopic spina bifida surgery**

**Aim of the study**

**To survey fetal surgeons with experience in prenatal spina bifida aperta surgery in order to determine the face and content validity of a high-fidelity simulation model for fetoscopic spina bifida aperta surgery.**

**Background**

**While interest for fetoscopic spina bifida repair is growing, there is a need for training possibilities for the multidisciplinary operative team. The IDEAL recommendations for surgical innovation and evaluation, state that preclinical research using validated simulation training models is essential prior to first in human trials.**^1,2^ **Such models may help in assessing the learning curve and competency, overcoming the initial learning curve, and avoiding or limiting training during clinical procedures.**^3,4^ **We therefore propose a high-fidelity^5^ simulation model for fetoscopic spina bifida surgery.**

**Description of the high-fidelity model**

We created a high-fidelity model **for fetoscopic spina bifida surgery** based on an analysis of the operative steps and conditions present when doing a layered prenatal repair.

Clinical procedure

The gestational age at fetal repair typically is around 24 weeks of gestation.^6-8^ At that time the fetal weight is 662±77g^9^ and the abdominal circumference 187±10mm^9^. The anatomical region of the interest is in 95% of cases located in the lumbar region^6^. The current literature on open^6,10^ as well as fetoscopic repair^7,8,11^ describes several steps in a prenatal repair. We empirically broke the procedure into 10 consecutive steps (Table 1, left column).

Animal model

We propose to use the adult rabbit (estd. weight 3-4 kg), which has been previously used as a training model for other procedures in pediatric^12^ and fetoscopic^13^ surgery. Herein, its insufflated abdominal cavity serves as an amniotic cavity hence mimics the working space.^13^ The physiologically dilated stomach mimics the surgical target because its circumference (165±13mm) is close to the abdominal circumference of a human fetus at 22-24 weeks (Figure 1).^14^

The rabbits are put under general anesthesia without intubation yet with insertion of a gastric tube. The 10 clinical steps are recapitulated in two phases: gastric fundoplication^15^ (8 steps) and suturing of a patch to the gastric wall (2 steps). These mimic fragile tissue manipulation, endoscopic dissection and suturing required for performing a two-layer watertight repair (Table 1, right column; Figures 2 and 3).

| Figure 1: Comparison of the clinical (fetal lower back) and simulated (rabbit gastric body) surgical target for spina bifida repair. Copyright by the authors. |
| --- |
| 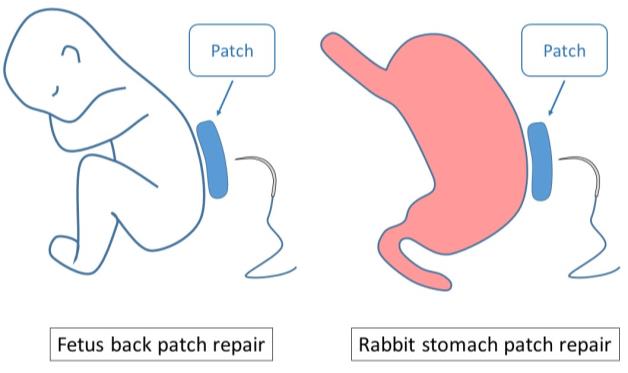 |

Figure 2 – Surgical set-up for simulated fetoscopic spina bifida repair using the in vivo rabbit model. In this particular experiment a single-port access was used as well as recording of surgical motions. Copyright by the authors.

**
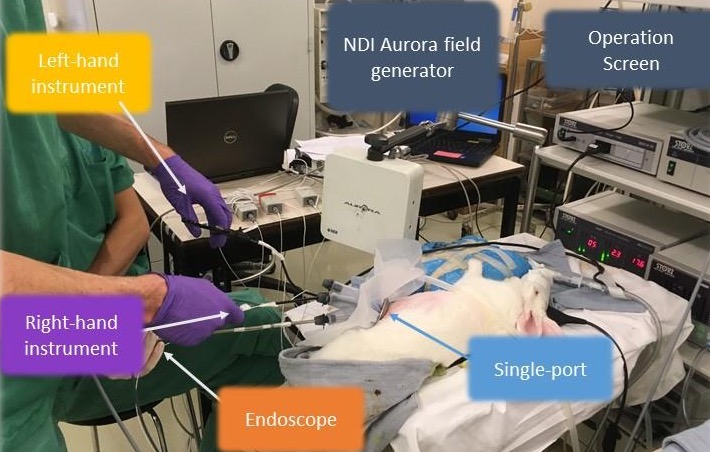
**

Table 1 – Comparison of 10 steps in a clinical spina bifida fetoscopic repair as described by Belfort et al.^7^ and a simulated fetoscopic repair in the rabbit model

|  | **Surgical steps** | **Clinical fetoscopic repair** | **Simulated fetoscopic repair in the adult rabbit** |
| --- | --- | --- | --- |
| 1 | Uterine exposition | Exteriorization, exposition and keeping the uterus moistened | Right ¾ lateral position, gastric tube insertion, shaving of frontal abdominal wall and right hemi-thorax. |
| 2 | Uterine access | Transmyometrial membrane fixation, cannulated uterine access via Seldinger-technique under ultrasound guidance | Transabdominal peritoneal fixation, visually-controlled open primary cannula insertion, and Seldinger-technique for secondary cannulas. |
| 3 | Creating workspace | CO_2_ pneumamnion | CO_2_ pneumoperitoneum |
| 4 | Exposition of target area | Fetal manipulation by instruments to provide access to the lumbar region | Exposition of esophago-gastric junction (EGJ) by manipulation and division of triangular ligaments and folding left and median liver lobes away of EGJ |
| 5 | Dissection | Dissection of the placode to completely untether it. | Dissection of the junction and the upper part of the gastrohepatic ligament. |
| 6 | Tissue resection and undermining | Circumferential resection of the junction line and undermining of the skin. | Undermining of the gastric fundus by division of the gastro-peritoneal ligament, from the EGJ and to the spleen. |
| 7 | Tissue mobilization | Approximation of lumbar skin edges | Pulling of the gastric fundus behind the EGJ to create a fundoplication valve. |
| 8 | Closure of the first layer | Dissection and suturing of myo-fascial flaps with/without patch | Nissen fundoplication using 4 intra-corporeal sutures^15^ |
| 9 | Closure of the second layer | Fetal skin closure with running sutures. | Simulated skin closure by suturing a patch on the anterior wall of the stomach with running sutures. |
| 10 | Quality assessment of the repair | Quality assessment of the skin suture line by inspection and adjustment. | Inspection of the patch suture line and adjustment.* |

* The quality of the suture line is later assessed by a watertightness test under 30cmH_2_O^16^.

Figure 3 – Comparison of essential steps performed during a clinical fetoscopic two-layer spina bifida repair (A-E) and simulated fetoscopic repair in the rabbit model (F-J). Copyright by the authors.


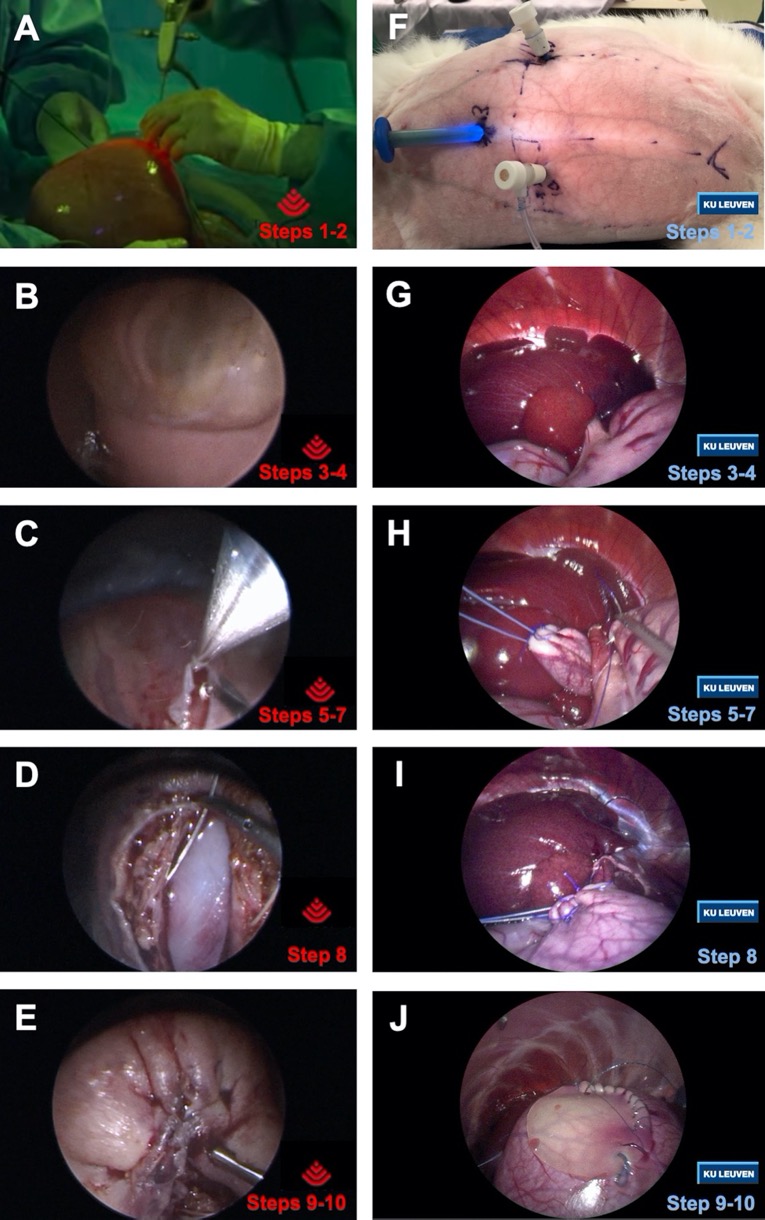


# **Survey**

# **Fetal surgeon demographic Information**

1. In which **continent** do you practice?

- **Asia**
- **North America**
- **South America**
- **Australia**
- **Europe**

1. **What is your specialty?**

- **Obstetrician-gynecologist**
- **(Pediatric) neurosurgeon**
- **Pediatric surgeon**

1. **How many years of experience** do you have since you finished your general medical training curriculum?

|  | **Number of years of experience** |
| --- | --- |
| As a specialist or specialist in training |  |
| As a laparoscopic surgeon |  |

1. What is your **handedness?**

- **Right-Handed**
- **Left-Handed**

1. **How many** fetal spina bifida repairs have you performed in humans as first surgeon?

| **Approach** | **As first surgeon** | | | | |
| --- | --- | --- | --- | --- | --- |
| Open | 1-30 | 31-60 | 61-90 | 91-120 | ≥121 |
| Fetoscopy | 1-30 | 31-60 | 61-90 | 91-120 | ≥121 |

1. **How many** fetal spina bifida repairs have you performed in humans as second surgeon?

| **Approach** | **As second surgeon** | | | | |
| --- | --- | --- | --- | --- | --- |
| Open | 1-30 | 31-60 | 61-90 | 91-120 | ≥121 |
| Fetoscopy | 1-30 | 31-60 | 61-90 | 91-120 | ≥121 |

1. Have you ever **trained on a simulator** for fetal spina bifida repair and, if yes **how many simulations** have you done?

| **Simulator** | **Virtual Reality** | **Box Trainer** | **Animal model** | **Human cadaver** |
| --- | --- | --- | --- | --- |
| For the open approach | - 1-30  - 31-60  - 61-90  - 91-120  - ≥121 | idem | idem | idem |
| For the fetoscopic approach | Idem | idem | idem | idem |

# **VALIDATION questionnaire**

- **Face validity of simulation model**

**Definition: This describes the realism of the simulator.^3,17^**

1. **Realism of the Surgical Target**: How realistic is **the dilated rabbit stomach to mimic the lumbar region of a human fetus?**

| **Realism** | 1) Absolutely not realistic | 2)  Not realistic | 3) Somewhat not realistic | 4) Undecided | 5) Somewhat realistic | 6)  Realistic | 7) Absolutely realistic |
| --- | --- | --- | --- | --- | --- | --- | --- |

1. **Realism of the Surgical Cavity**: How realistic is **the abdominal cavity to mimic the amniotic cavity?**

| **Realism** | 1) Absolutely not realistic | 2)  Not realistic | 3) Somewhat not realistic | 4) Undecided | 5) Somewhat realistic | 6)  Realistic | 7) Absolutely realistic |
| --- | --- | --- | --- | --- | --- | --- | --- |
| **in terms of surgical workspace** |  |  |  |  |  |  |  |
| **in terms of vision** |  |  |  |  |  |  |  |

1. **Realism of the Instrumentation set:** How realistic are the surgical instruments to mimic what is clinically used?

| **Realism of** | 1) Absolutely not realistic | 2)  Not realistic | 3) Somewhat not realistic | 4) Undecided | 5) Somewhat realistic | 6)  Realistic | 7)  Absolutely realistic |
| --- | --- | --- | --- | --- | --- | --- | --- |
| **Endoscope** |  |  |  |  |  |  |  |
| **Grasping forceps** |  |  |  |  |  |  |  |
| **Scissors** |  |  |  |  |  |  |  |
| **Coagulating and dissecting hook** |  |  |  |  |  |  |  |
| **Dissector** |  |  |  |  |  |  |  |
| **Needle holders** |  |  |  |  |  |  |  |

1. **Realism of surgical scene:** How realistic are the animal positioning, the position of the video monitor and the surgeons around the operation table to mimic the conditions clinically present?

| **Realism of** | 1)  Absolutely not realistic | 2)  Not realistic | 3) Somewhat not realistic | 4) Undecided | 5)  Somewhat realistic | 6) Realistic | 7) Absolutely realistic |
| --- | --- | --- | --- | --- | --- | --- | --- |
| Animal positioning |  |  |  |  |  |  |  |
| **Position of the video monitor** |  |  |  |  |  |  |  |
| **Position of the 1^st^ surgeon** |  |  |  |  |  |  |  |
| **Position of the 2^nd^ surgeon** |  |  |  |  |  |  |  |

1. **Realism of the 10 surgical steps** (table 1)

| **Realism of** | 1) Absolutely not realistic | 2)  Not realistic | 3)  Somewhat not realistic | 4) Undecided | 5)  Somewhat realistic | 6)  Realistic | 7) Absolutely realistic |
| --- | --- | --- | --- | --- | --- | --- | --- |
| **Exposition** |  |  |  |  |  |  |  |
| **Port insertion** |  |  |  |  |  |  |  |
| **Insufflation** |  |  |  |  |  |  |  |
| **Fetal positioning** |  |  |  |  |  |  |  |
| **Dissection** |  |  |  |  |  |  |  |
| **Resection** |  |  |  |  |  |  |  |
| **Mobilization** |  |  |  |  |  |  |  |
| **Patch** |  |  |  |  |  |  |  |
| **Skin** |  |  |  |  |  |  |  |
| **Quality assessment** |  |  |  |  |  |  |  |

1. **Realism of depth perception**: **How realistic is overall depth** perception to mimic clinical conditions?

| **Realism** | 1)  Absolutely not realistic | 2)  Not realistic | 3) Somewhat not realistic | 4) Undecided | 5) Somewhat realistic | 6)  Realistic | 7) Absolutely realistic |
| --- | --- | --- | --- | --- | --- | --- | --- |

- **Content validity of simulation model**

**Definition: characterizes the usefulness and appropriateness of the content of simulator as a teaching modality.^3,17^**

1. **Usefulness and appropriateness of instrument handling: t**he simulator gives the trainee the opportunity to improve her/his instrument handling skills.

| **Usefulness** | 1) Strongly Disagree | 2) Disagree | 3) Neutral | 4) Agree | 5) Strongly Agree |
| --- | --- | --- | --- | --- | --- |

1. **Usefulness and appropriateness of suturing: t**he simulator provides the trainee with the opportunity to improve her/his suturing skills.

| **Usefulness** | 1) Strongly Disagree | 2) Disagree | 3) Neutral | 4) Agree | 5) Strongly Agree |
| --- | --- | --- | --- | --- | --- |

1. **Usefulness and appropriateness for self-confidence: t**he simulator provides the trainee with overall confidence.

| **Usefulness** | 1) Strongly Disagree | 2) Disagree | 3) Neutral | 4) Agree | 5) Strongly Agree |
| --- | --- | --- | --- | --- | --- |

1. **Usefulness and appropriateness of the 10 surgical steps:** The simulator helps the trainee understand **fetoscopic spina bifida repair** and train for **it.**

| **Usefulness** | 1) Strongly Disagree | 2) Disagree | 3) Neutral | 4) Agree | 5) Strongly Agree |
| --- | --- | --- | --- | --- | --- |
| **Exposition** |  |  |  |  |  |
| **Port insertion** |  |  |  |  |  |
| **Insufflation** |  |  |  |  |  |
| **Fetal positioning** |  |  |  |  |  |
| **Dissection** |  |  |  |  |  |
| **Resection** |  |  |  |  |  |
| **Mobilization** |  |  |  |  |  |
| **Patch** |  |  |  |  |  |
| **Skin** |  |  |  |  |  |
| **Quality assessment** |  |  |  |  |  |

1. **Overall usefulness and appropriateness of the simulator**

| **Overall usefulness** | 1) Strongly Disagree | 2) Disagree | 3) Neutral | 4) Agree | 5) Strongly Agree |
| --- | --- | --- | --- | --- | --- |
| **The simulated fetoscopic repair is as difficult as the procedure in humans.** |  |  |  |  |  |
| The **simulated fetoscopic repair** expose the trainee to a stress similar to that in the clinical operating room. |  |  |  |  |  |
| Would you recommend this simulator for training for fetoscopic spina bifida repair? |  |  |  |  |  |

**Please feel free to add any remarks to improve the simulator:**

**References**

- - 1 McCulloch, P. *et al.* No surgical innovation without evaluation: the IDEAL recommendations. *Lancet* **374**, 1105-1112, doi:10.1016/S0140-6736(09)61116-8 (2009).
  - 2 Hirst, A. *et al.* No Surgical Innovation Without Evaluation: Evolution and Further Development of the IDEAL Framework and Recommendations. *Annals of surgery* **269**, 211-220, doi:10.1097/SLA.0000000000002794 (2019).
  - 3 Carter, F. J. *et al.* Consensus guidelines for validation of virtual reality surgical simulators. *Surg Endosc* **19**, 1523-1532, doi:10.1007/s00464-005-0384-2 (2005).
  - 4 Schout, B. M., Hendrikx, A. J., Scheele, F., Bemelmans, B. L. & Scherpbier, A. J. Validation and implementation of surgical simulators: a critical review of present, past, and future. *Surg Endosc* **24**, 536-546, doi:10.1007/s00464-009-0634-9 (2010).
  - 5 Tan, S. S. & Sarker, S. K. Simulation in surgery: a review. *Scott Med J* **56**, 104-109, doi:10.1258/smj.2011.011098 (2011).
  - 6 Adzick, N. S. *et al.* A randomized trial of prenatal versus postnatal repair of myelomeningocele. *N Engl J Med* **364**, 993-1004, doi:10.1056/NEJMoa1014379 (2011).
  - 7 Belfort, M. A. *et al.* Fetoscopic Open Neural Tube Defect Repair: Development and Refinement of a Two-Port, Carbon Dioxide Insufflation Technique. *Obstetrics and gynecology* **129**, 734-743, doi:10.1097/AOG.0000000000001941 (2017).
  - 8 Lapa Pedreira, D. A. *et al.* Percutaneous fetoscopic closure of large open spina bifida using a bilaminar skin substitute. *Ultrasound in obstetrics & gynecology : the official journal of the International Society of Ultrasound in Obstetrics and Gynecology* **52**, 458-466, doi:10.1002/uog.19001 (2018).
  - 9 Salomon, L. J., Bernard, J. P. & Ville, Y. Estimation of fetal weight: reference range at 20-36 weeks' gestation and comparison with actual birth-weight reference range. *Ultrasound in obstetrics & gynecology : the official journal of the International Society of Ultrasound in Obstetrics and Gynecology* **29**, 550-555, doi:10.1002/uog.4019 (2007).
  - 10 Heuer, G. G., Adzick, N. S. & Sutton, L. N. Fetal myelomeningocele closure: technical considerations. *Fetal diagnosis and therapy* **37**, 166-171, doi:10.1159/000363182 (2015).
  - 11 Ovaere, C. *et al.* Prenatal diagnosis and patient preferences in patients with neural tube defects around the advent of fetal surgery in Belgium and Holland. *Fetal diagnosis and therapy* **37**, 226-234, doi:10.1159/000365214 (2015).
  - 12 Luks, F. I., Peers, K. H., Deprest, J. A. & Lerut, T. E. Gasless laparoscopy in infants: the rabbit model. *Journal of pediatric surgery* **30**, 1206-1208, doi:10.1016/0022-3468(95)90023-3 (1995).
  - 13 Quintero, R. A. *et al.* Hydrolaparoscopy in the rabbit: a fine model for the development of operative fetoscopy. *Am J Obstet Gynecol* **171**, 1139-1142, doi:10.1016/0002-9378(94)90052-3 (1994).
  - 14 Chitty, L. S., Altman, D. G., Henderson, A. & Campbell, S. Charts of fetal size: 3. Abdominal measurements. *British journal of obstetrics and gynaecology* **101**, 125-131, doi:10.1111/j.1471-0528.1994.tb13077.x (1994).
  - 15 Jamieson, G. G., Watson, D. I., Britten-Jones, R., Mitchell, P. C. & Anvari, M. Laparoscopic Nissen fundoplication. *Annals of surgery* **220**, 137-145, doi:10.1097/00000658-199408000-00004 (1994).
  - 16 Avery, R. A. *et al.* Reference range for cerebrospinal fluid opening pressure in children. *N Engl J Med* **363**, 891-893, doi:10.1056/NEJMc1004957 (2010).
  - 17 Dawson, B. & Trapp, R. G. in *Basic and Clinical Biostatistics* *biostatistics* (eds B. Dawson & R.G. Trapp) Ch. 11, 287-289 (McGraw-Hill Companies, 2004).
